# Mind the Gap Between Estimated Needs and Current Resources in Chronic Kidney Disease

**DOI:** 10.3390/healthcare13222826

**Published:** 2025-11-07

**Authors:** Francesca K. Martino, Federico Nalesso

**Affiliations:** Nephrology, Dialysis, Transplantation Unit, Department of Medicine (DIMED), University-Hospital of Padua, 35128 Padova, Italy; federico.nalesso@unipd.it

**Keywords:** chronic kidney disease, prevalence, nephrology care, human resources, facilities

## Abstract

**Background/Objectives:** In recent decades, chronic kidney disease (CKD) has increased its prevalence in the general population, reaching about 10%. The increasing number of older patients and the expansion of treatment options could necessitate a growing number of nephrologists/nurses. This report aims to estimate the resources required to manage the CKD population and hypothesize possible strategies to address the discrepancy between the estimated need and available resources. **Methods:** Based on previous reports about CKD in our geographic area and the data reported by the Italian Statistics Institute (ISTAT), we estimated the number of patients who should be referred to a nephrologist in our district, and consequently the number of nephrology consults and the need of nephrologists (considering 14 visits for 5 days/week, 48 weeks/year for each nephrologist). **Results:** The Padua district has approximately 240,257 inhabitants aged 40 years and older. Of these, 31,139 are estimated to have CKD, and 540 are potentially affected by CKD stage 5. The estimated number of outpatient visits is approximately 178 per day, which would require 12 full-time employed nephrologists. On the contrary, only two nephrologists are currently involved full-time in outpatient clinics, while eight are involved part-time. Finally, 540 CKD G5 surpasses the current available dialysis seats (184 hemodialysis seats and 80 peritoneal dialysis seats). **Conclusions:** In the Padua Healthcare district, the estimated outpatient clinic demand and the potential need for renal replacement therapy exceed human and facility resources, indicating a need for nephrologists in outpatient clinics that is more than four to six times the current numbers. This discrepancy highlights the need for a multidimensional approach that promotes active collaboration with general practice, telemedicine, and an informative campaign targeting the population.

## 1. Introduction

In recent decades, the prevalence of chronic kidney disease (CKD) has been on a steady rise, mirroring the aging of the global population. This trend has transformed CKD into a global health concern. The primary reason for this concern is the profound impact of CKD on cardiovascular risk, significantly worsening the prognosis and survival of patients [1,2,3,4]. Notably, CKD is the 11th leading cause of death in the general population worldwide [3]. Furthermore, it serves as a risk factor for cardiovascular disease, specifically, both albuminuria and low estimated glomerular filtration rate (eGFR) escalate the risk of major cardiovascular death and events [5,6]. This global impact underscores the need for a coordinated response to manage CKD effectively.

Epidemiological data from around the world paint a concerning picture, with an estimated 697.5 million patients and a global prevalence of approximately 9.1% [7]. Although there were described disparities in CKD prevalence and burden between low-, middle-, and high-income countries, the increasing rate of CKD highlights the need for a coordinated response. This response should include a local evaluation of CKD distribution to effectively manage the expected requirement for nephrological care. The differences in CKD prevalence are likely related to the inhabitants’ age, comorbidities, socioeconomic characteristics, and the country’s capacity to diagnose CKD [8,9]. Disparities in the prevalence of chronic kidney disease (CKD) exist even in high-income countries. For example, in the United States, the estimated prevalence of CKD is around 14% [10], while in Canada, it is approximately 10% [11]. In Japan, the prevalence is similarly noted [12]. In Western Australia, the age- and sex-standardized prevalence is about 6% [13]. In Europe, the adjusted prevalence of CKD stages G1-5 varies significantly, ranging from 3% to 20% across different regions [14]. European countries exhibit a disparity in comorbidities such as diabetes, hypertension, and obesity, but they also demonstrate different lifestyle habits, including levels of physical activity, smoking, and salt intake. All these factors can justify a high variability in different nations, with the lowest prevalence in Norway, where it is around 3% [15], slightly higher in the Spanish population, with 4.9% [16,17], significantly higher prevalence was estimated in Ireland with 11.8% according to their health system [18], even higher in the United Kingdom considering the CKD G3-5 prevalence of 11.3% [19] and in Germany, where the prevalence varies between 18 and 21% according to region [20].

Even within the same country, such as Italy, the estimation of CKD prevalence varies across different regions of the peninsula. In 2010, Gambaro et al. assessed the prevalence of CKD at 12.7% among 6200 northeast inhabitants aged over 40 years in the INCIPE study (Initiative on Nephropathy, of relevance to public health, which is Chronic, possibly in its Initial stages, and carries a Potential risk of major clinical Endpoints) [21]. However, in the same year, Guarda et al. reported a lower prevalence of CKD, ranging from 3.5% to 6%, according to gender and the subdistrict in the province of Mantua [22]. Another significant disparity in prevalence was referred in central of Italy, where Gorini et al. in 2012 evaluated the prevalence of CKD around 24.6%, in 573 healthy volunteers aged 21–62 years (FARM study) [23], while other three observational studies reported significant lower prevalence: Donfrancesco et al. in 2012 describes in MATISS study a prevalence of 1% in the men and of 1.6% in females in low risk of population [24], Ciao G et al. in 2014 found CKD prevalence in Umbria around 1% on 50,000 inhabitants [25], and Marino et al. in 2020 showed a prevalence of CKD G1-5 in the Lazio region of 1.76% [26]. Furthermore, in 2012, Capuano et al. reported different CKD prevalence rates in males and females, which were 6.2% and 4.5%, respectively, in southern Italy [27]. Pani et al. estimated the prevalence of CKD to be around 15% in residents of eastern Sardinia in the SardiNIA study [28]. Finally, in 2015, De Nicola et al. estimated the prevalence of CKD in 7% of 9020 people aged between 35 and 79 years from 20 Italian regions (CAREHES: Cardiovascular risk in Renal Patients of the Italian Health Examination Survey) in a large-scale survey across Italy [29].

The significant contrasts in CKD prevalence may be attributed only in part to comorbidity rates and lifestyle habits, considering the uniformity of the national healthcare system and lifestyle patterns across the country. The larger disparities between the reports may be easily explained by the differing criteria used for CKD diagnosis. Some of these reports considered the national health system’s code for the diagnosis of CKD [22,26,30], which includes criteria that may underestimate the actual number of patients being linked to the code release by nephrologists. In contrast, others referred to a random sample of the entire population but used different formulas in estimating eGFR [23,27], while others analyzed all individuals within a specific area, taking into account access to nephrological facilities. This geopartized prevalence in different regions of Italy highlights the importance of local CKD estimation and the criteria for defining CKD status [31], recognizing the need for nephrological care and the development of tailored management strategies to support CKD patients in their respective districts. To evaluate an appropriate nephrological intervention in the local health system, it is essential to consider the implementation of standards of care over the last decade, which align with new guidelines [32,33] and the availability of new drug classes in nephrological care [32,33]. The treatment of kidney disease requires lifestyle interventions (such as implementing physical activity [34,35,36], improving diet habits with a low-protein diet [37,38,39,40,41], and limited salt intake [42,43]), and the optimization of hypertensive and diabetes care (such as the use of sodium-glucose cotransporter-2 (SGLT2) inhibitors in patients with and without diabetes [44,45,46,47,48,49], mineralocorticoid receptor antagonist (MRA) [50,51], and glucagon-like peptide-1 receptor agonists (GLP-1 RA) in adults with type 2 diabetes [52]). All these procedures require extensive access to nephrology facilities for diagnosis, treatment, and monitoring of CKD patients.

In the context of the request gain, it is reasonable to estimate the number of patients who require a nephrologist’s consultation to organize nephrology facilities, such as outpatient clinics, according to the actual needs in our district. Our report aims to estimate the number of patients in our district based on the previous prevalence results, and to determine the nephrology care needs in our area, providing data to inform healthcare planning and resource allocation for CKD management. Furthermore, it offers an interesting perspective focusing on the disparity between limited resources and the greater need in nephrological care.

## 2. Materials and Methods

### 2.1. Inhabitants Determination

The number of patients referred to the Padua Healthcare district was determined by analyzing data from the adult population over 40 years old from all municipalities in the district, as reported by the Italian Statistics Institute (ISTAT) as of 1 January 2024 (available on the website https://www.tuttitalia.it/statistiche/popolazione-eta-sesso-stato-civile-2024, accessed 15 April 2025). Specifically, in the Padua Healthcare District, there are 17 municipalities (Abano Terme, Albignasego, Cadoneghe, Cervarese, Santa Croce, Limena, Maserà, Mestrino, Montegrotto, Padua, Rovolon, Rubano, Saccolongo, Selvazzano Dentro, Teolo, Torreglia, Veggiano).

### 2.2. Choice of Epidemiologic Studies to Determine CKD Prevalence

Considering the earlier studies about CKD prevalence and the disparity in the prevalence, we decided to refer to the INCIPE study. The principal reason to support our choice is the referent population; the INCIPE study was conducted in the Veneto region, where the Padua Healthcare district is located. Although data collection took place in 2006, which may seem somewhat outdated, there are no more recent studies on this topic available in our area. Furthermore, we emphasize the data collection periods of the other main epidemiological reports in Italy. Specifically, the CAREHES study was conducted between 2008 and 2012, whereas the SardiNIA study, a cohort study, began in 2001 and had an average follow-up period of 7 years. Consequently, these epidemiological studies do not significantly narrow the temporal gap between their observation periods and the present time in estimating the number of CKD patients. Therefore, pending new epidemiological studies, which are strongly recommended, we refer to the INCIPE study, as it is thought to represent our district population better. Furthermore, the INCIPE study results enabled the estimation of albuminuria in CKD G1-4, allowing for stratification of the risk of CKD progression and the suggested number of nephrological controls. As reported in the supplemental material of the INCIPE study [21], we have the data to calculate the prevalence in CKD G1-4.

Unfortunately, the INCIPE study did not report the prevalence of CKD G5. The absence of CKD G5 data has been addressed by utilizing the data from the Vicenza Study on CKD G5, published in 2024. This study, which focused on the Veneto population in 2020, provided the missing data. Specifically, the comparable characteristics of the population and the considered period make the study results reliable for estimating CKD G5 in the Padua district.

### 2.3. Statistical Analysis

#### Operative Procedures for the Estimation of CKD Prevalence According to eGFR and Albuminuria

We estimated the number of CKD patients for each specific group (G1, G2, G3, G4) according to estimated Glomerular Filtration Rate (eGFR) based on the prevalence reported in the INCIPE study, which provided a comprehensive analysis of CKD G1-4 prevalence by age class (Table 1).

Furthermore, the risk of CKD progression was assessed using the results of the INCIPE study. Specifically, we stratified the population according to the stages G1–G4; subsequently, we divided the prevalence of each stage into three albuminuria categories (<30 mg/g, 30–299 mg/g, and >300 mg/g). Specifically, since albuminuria grading in CKD G3 was reported for the entire group, we divided the number of patients, considering a ratio of prevalence of CKD G3a/G3b of 5:1, to estimate the risk of CKD progression at this stage. Table 2 reports the prevalence of CKD G1-4 and albuminuria grading.

Finally, we assessed the prevalence according to the CKD G5 Vicenza study [53], considering that all CKD G5 patients are high-risk patients; we did not split the population for albuminuria grading.

Furthermore, according to the CKD G5 Vicenza study results, we divided CKD G5 individuals into two categories: those who require renal replacement therapy (37.19%, including hemodialysis and peritoneal dialysis), and patients who do not require dialysis (43.6%, including predialysis programs, long-term conservative management, and non-followed patients). Notably, the CKD G5 Vicenza study reported a prevalence of 19.2% among patients without nephrological care. Table 3 reports the age distribution in CKD G5.

### 2.4. Estimation of the Number of Nephrologists Controls

We calculated the number of outpatient clinic visits in one year based on the estimated number of patients in our district and their risk of CKD progression, as reported in KDIGO guidelines, as shown in Table 4.

For example, in a patient with eGFR equal to 52 who had also albuminuria equal to 220 mg/g, Creatinine merits a nephrological follow-up at least every six months to assess the rate of kidney disease progression, to correct the possible risk factor, and to optimize the treatment.

Concurrently, we estimated the number of nephrologists needed in one year. Considering the current arrangement between specialists and the district hospital, which refers to 38 h per week, 48 weeks per year, we deemed adequate for each full-time employed nephrologist to take into account the following:

- 14 visits per day, five days a week, 48 weeks a year.
- 30 min for each consultation.
- An average period of 3 h per week, related to other management activities related to the outpatient clinic.

It is essential to note that we did not include patients undergoing renal replacement therapy in our calculations, as they are typically referred to dialysis facilities for treatment.

## 3. Results

**Figure 1:**
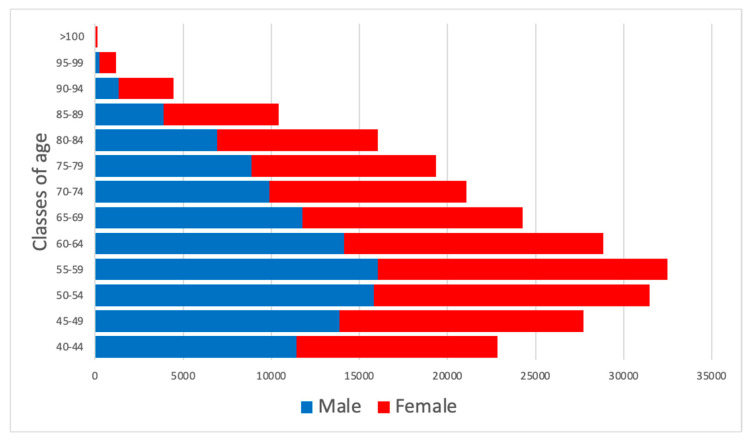
The diagram represents the entire population in the Padua Healthcare District, categorized by gender. The red boxes represent females, and the blue boxes represent males.

The Padua district had an adult population over 40 years old of 240,257, according to the ISTAT report as of 1 January 2024. 114,201 were male and 126,056 were female. Figure 1 presents the gender distribution by age class.

**Figure 2:**
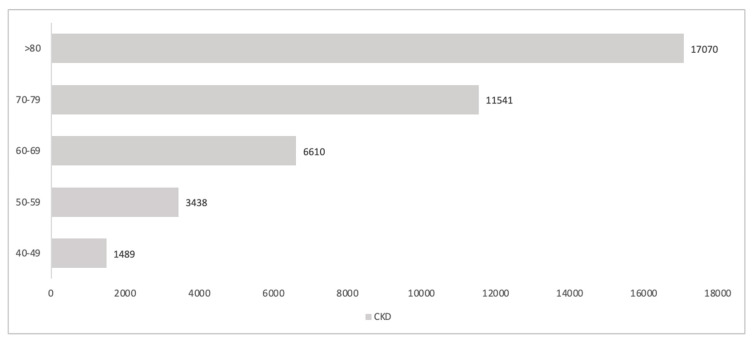
Projection of CKD G1-5 by age classes in the Padua healthcare district.

The estimated number of inhabitants over 40 years old with CKD G1-5 was 31,139, of whom 16,369 (52%) were male. Figure 2 reports the estimation of the age distribution of the CKD population in the Padua Healthcare district, with a cumulative number of 40,148.

Based on CKD G1-5 estimation according to age classes, a higher number of CKD patients are in the age group over 70 years old, which represents approximately 70% of the population.

### 3.1. CKD G1

**Figure 3:**
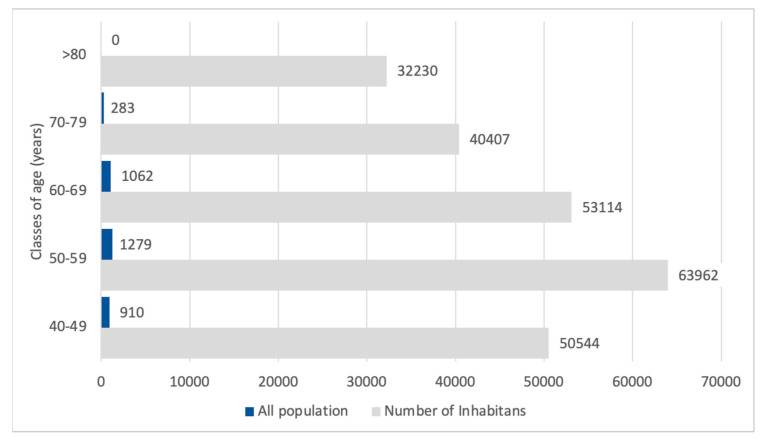
Estimated CKD G1 population by age classes in comparison with the whole population in the Padua Healthcare district. CKDG1 patients are represented in blue boxes, while the gray boxes represent the inhabitants of the Padua Healthcare District.

The estimated number of inhabitants with CKD G1 in the entire population was 4084, while it was 4463 according to the classes of age and gender. The higher prevalence of CKD G1 patients was observed in age classes between 50 and 69, which represents over 65% of all CKD G1 patients—Figure 3 reports the age distribution.

**Figure 4:**
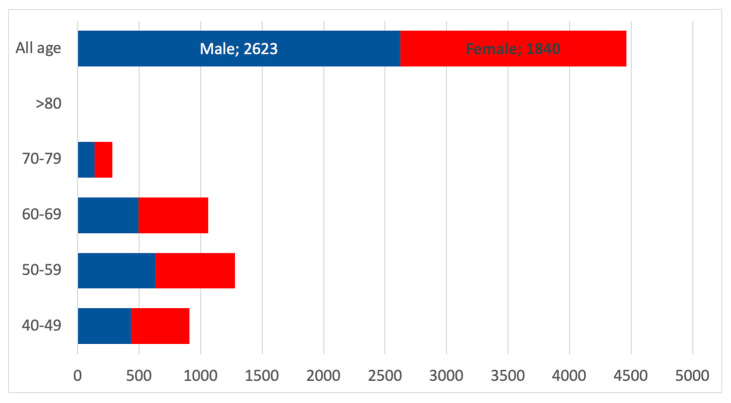
Estimated CKD G1 population according to gender and age classes in comparison with all CKD patients. The red boxes represent female patients with CKD G1, and the blue boxes represent males with CKD G1.

Finally, we estimated the number of CKD G1 patients by age class and gender, as shown in Figure 4. Notably, the projection in our population shows that the higher number of patients should be female and in the middle age classes, between 50 and 69 years. Likely, the prevalence of CKD G1 should be similar in both genders, considering the little difference in prevalence between males and females reported in the INCIPE study, at 1.7% and 1.6%, respectively.

### 3.2. CKD G2

**Figure 5:**
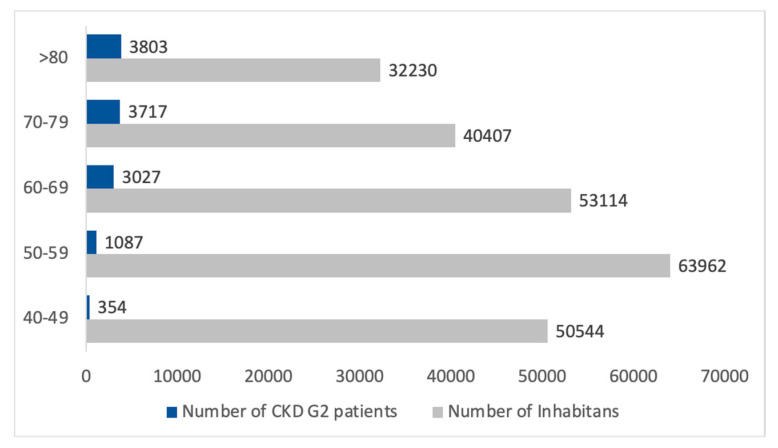
Estimated CKD G2 population by age classes in comparison with the entire population in the Padua Healthcare district. CKD G2 patients are represented in blue boxes, while the gray boxes represent the inhabitants of the Padua Healthcare District.

The estimated number of inhabitants with CKD G2 was 10,331, according to the INCIPE study results. The higher prevalence of CKD G2 patients was in age classes over 70 years, which represents over 72% of the estimated G2 population in the Padua Healthcare district. Figure 5 reports the age classes distribution.

**Figure 6:**
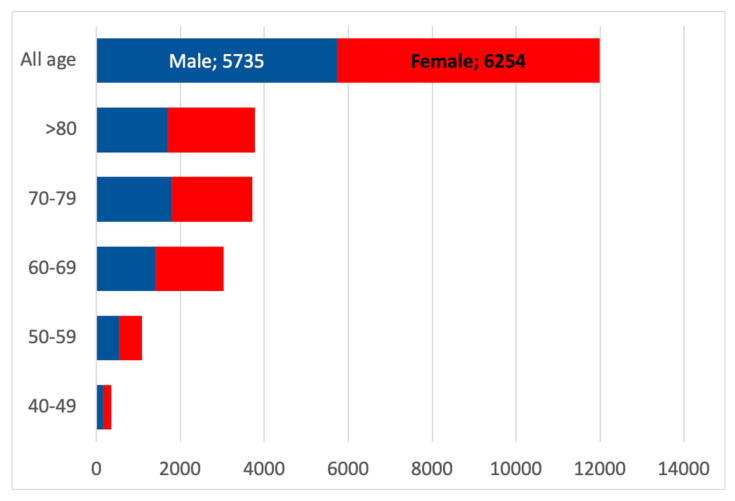
Estimated CKD G2 population according to gender and age classes. The red boxes represent female patients with CKD G2, and the blue boxes represent males with CKD G2.

The estimation of the number of patients with CKD G2, categorized by gender and age classes, was 11,989, indicating that the highest number of patients is over 70 years old, among whom the prevalence of CKD G2 reached 67%, as shown in Figure 6. Notably, the higher number of female patients in all age groups of CKD G2 is almost related to the gender distribution in the Padua Healthcare district.

### 3.3. CKD G3

**Figure 7:**
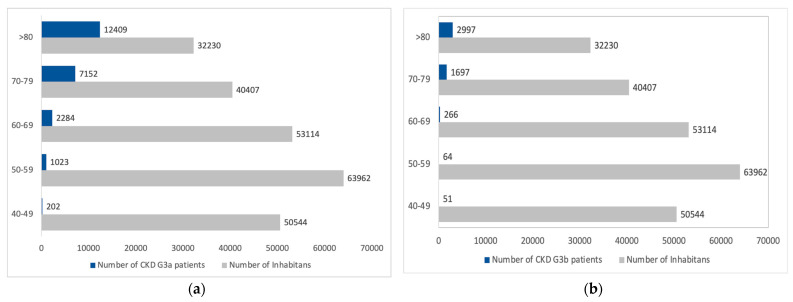
(**a**) Estimated CKD G3a prevalence by age classes; (**b**) Estimated CKD G3b prevalence by age classes. The gray boxes represent the inhabitants of the Padua Healthcare District. Blue boxes represent CKD G3 patients.

The estimated number of inhabitants with CKD G3 was 15,376 in the total population, based on the 6.4% prevalence reported in the INCIPE study. However, the estimation of CKD G3a and G3b, considering each age class and gender, amounts to 23,070 and 5075, respectively. The most prevalent patients in Padua Healthcare District should be the elderly patients over 70, representing over 80% of CKD G3 patients. Figure 7 presents the age distribution of CKD G3a and CKD G3b, as reported in the INCIPE study.

**Figure 8:**
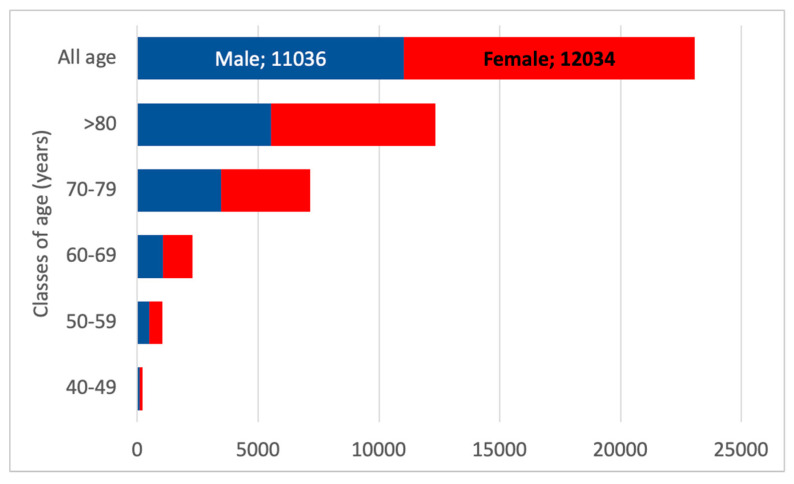
Estimated CKD G3 population according to gender. The red boxes represent female patients with CKD G3, and the blue boxes represent males with CKD G3.

Figure 8 illustrates the projection of CKD G3 according to age and gender classes. The number of females over 80 years old is expected to be the highest in the entire CKD G3 population in the Padua Healthcare district, as females represent 54% in the general population over 80 years old.

### 3.4. CKD G4

**Figure 9:**
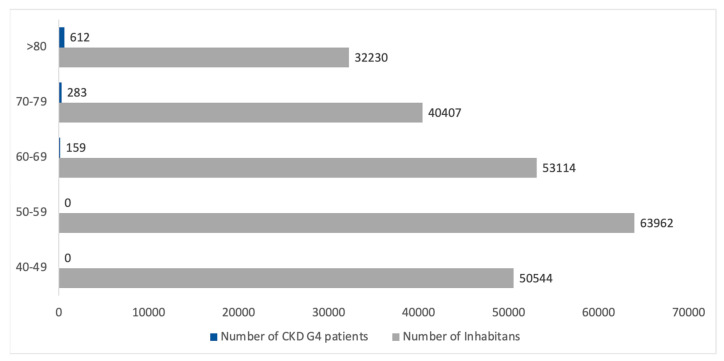
Estimated CKD G4 population by age classes in comparison with the entire population in the Padua Healthcare district. CKD G4 patients are represented in blue boxes, while the gray boxes represent the inhabitants of the Padua Healthcare District.

The estimated number of inhabitants with CKD G4 was 807 according to the INCIPE study. Again, the more prevalent CKD G4 patients were over 70 years old, which represents around 85% of all CKD G4 patients. Figure 9 presents the estimated age distribution for a total of 1055 patients. Notably, the number of patients in the young classes is poorly represented in the CKD G4 population.

**Figure 10:**
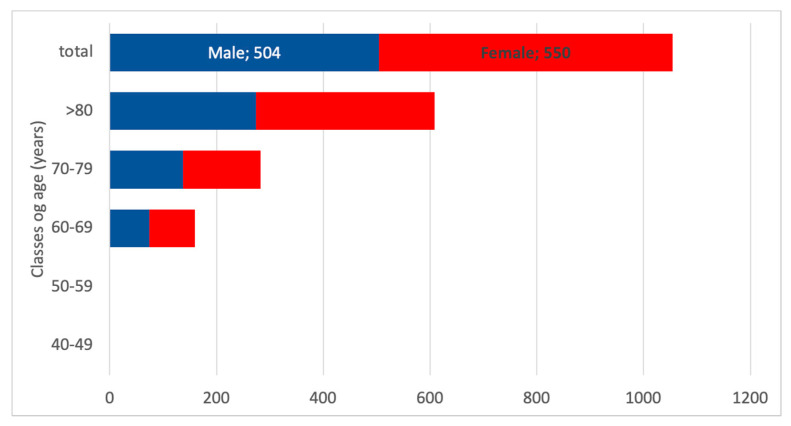
Estimated number of CKD G4 patients according to the classes of age and gender. The red boxes represent female CKD G4 patients, and the blue boxes the males with CKD G4.

Also in this case, the higher number of females is related to the higher number in the general population. Conversely, considering INCIPE results, the ratio between males and females is 3:2 in CKD G4 (Figure 10).

### 3.5. CKD G5

**Figure 11:**
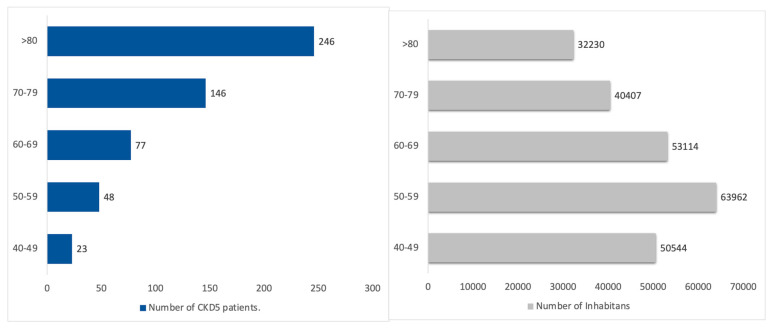
Estimated CKD G5 population by age classes, in the Padua Healthcare district, who are represented in blue boxes. In contrast, the gray boxes represent the inhabitants of the Padua Healthcare District.

The estimated number of inhabitants with CKD G5 was 540, according to the Vicenza CKD G5 study, of whom approximately 200 patients are expected to require renal replacement therapy. The estimated number of CKD G5 patients followed in outpatient clinics (conservative management, predialysis, and kidney transplant) is approximately 240. Furthermore, CKD G5 appears to be burdened by a notable prevalence of non-followed patients, which could be estimated at around 100 in the Padua Healthcare district. Figure 11 reports the age distribution of CKD G5.

**Figure 12:**
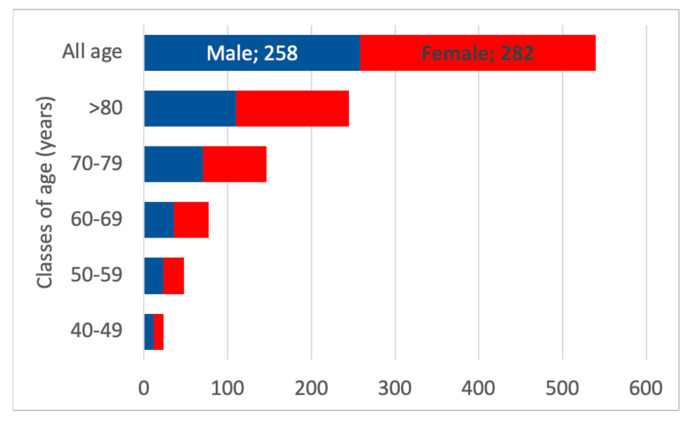
Estimated number of CKD G5 patients according to the classes of age and gender. The red boxes represent female patients with CKD G5, and the blue boxes represent males with CKD G5.

The gender distribution in CKD G5 is expected to be slightly skewed towards the female gender, as Figure 12 indicates.

### 3.6. Projection of the Number of Nephrologist Consultations in the Whole Population

The estimation of the risk progression in the CKD G1-5 Padua Healthcare District population revealed that 1006 patient (prevalence 0.4%) has a low risk of CKD progression, 21,215 (prevalence 8.8%) has a moderate risk, 6235 patients (2.6%) has a high risk, 2483 (1%) have a very high risk, and around 200 patients (0.08%) should need renal replacement therapy (RRT).

The projected number of the population according to the CKD stage and albuminuria grading exceeds 30,000 subjects, of whom only 1006 should be evaluated in a screening program. All other patients should undergo one or more annual controls, as shown in Table 5.

**Figure 13:**
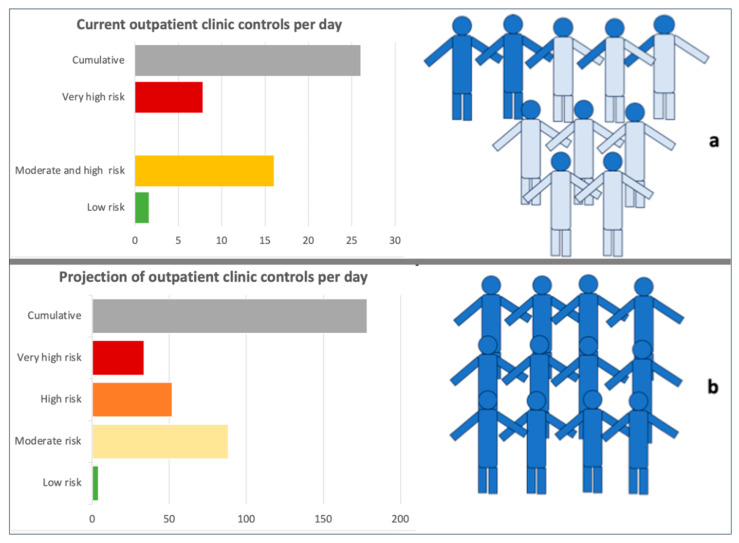
(**a**) Current and projected number of outpatient clinic controls according to risk stratification, (**b**) nephrologists’ need in the Padua District population. Dark blue represents the proportion of activity in the outpatient clinic for each nephrologist.

The estimated number of outpatient clinic controls by day could reach 178, assuming the need for at least twelve full-time employed nephrologists in the outpatient clinic, considering 14 consultations per day for each nephrologist. Conversely, in our district, the current number of nephrologists who follow outpatient clinic patients is approximately ten, of whom only two are full-time employed nephrologists in the outpatient clinic. Considering the outpatient clinic is part of other activities, such as dialysis management, inpatient clinics, and consultations in different departments. Figure 13a reports the current number of outpatient clinic controls per day and the number of nephrologists partially employed in the outpatient clinic, while Figure 13b reports the projection.

## 4. Discussion

The projection of epidemiological data on CKD in the Padua Healthcare District presents a concerning picture, with a vast number of patients to follow. Specifically, in our district, there should be over 30,000 subjects with CKD, of whom around twenty-one thousand have a low-moderate risk of CKD progression, while over eight thousand have a high or very high risk. The number of possible CKD patients would require twelve nephrologists, full-time employed in the outpatient clinics, to manage the treatment and reduce the risk of progression. On the contrary, only two nephrologists are currently full-time employees in the outpatient clinic, while the other eight are involved in various activities.

We selected the INCIPE [21] and CKD G5 Vicenza study [53] to enhance comparability between the Padua district population and those described in the study, given the limited difference in the timing of data collection between INCIPE and CAREHES or SardiNIA studies. In any case, if we refer to the CAREHES [29] or SardiNIA [28] studies, which reported the prevalence of CKD at 7.05% and 14.5%, respectively, we obtain 16,938 and 31,202 as the number of CKD G1-4 patients, and 123 and 179 daily controls, respectively.

Specifically, the CAREHES report [29] suggests a significantly lower number of patients with CKD and nephrology consultations, particularly in CKD G3. Still, the CAREHES study did not account for the prevalence of G1A1 and G2A1, and it did not take into account patients over 80 years old, who are numerically increasing in the decades, and who have the highest prevalence of severe CKD. The explanations for these disparities are likely related to various factors, including differences in population characteristics such as comorbidities, lifestyle habits, and age class. Specifically, the prevalence of diabetes, hypertension, and obesity is 31%, 21.9%, and 16.8% in CKD patients, according to the INCIPE study [21]; while CAREHES reported a higher prevalence of hypertension, around 78%, and obesity at 38.3% [29]. Furthermore, the INCIPE study did not take into account lifestyle habits like smoking and alcohol consumption, which could have a significant impact on the development of CKD [54,55,56,57,58,59].

However, the projection according to the SardiNIA study [28] shows a number similar to that of the INCIPE study for the number of CKD patients and outpatient clinic controls, but with a higher prevalence of CKD G1A1 and CKD G2A1 compared to the INCIPE study. Specifically, in the SardiNIA study the prevalence of low-risk patients was 84.6%, of moderate risk patients was 11.5%, of high-risk patients was 3.6%, and of very high-risk patients was 0.4%, while the INCIPE study estimated as 0.4% the low-risk category, as 8.8% the moderate-risk category, as 2.6% the high risk, and as 1% the very high risk.

Finally, the difference in prevalence could be attributed to the use of the MDRD formula for evaluating eGFR, rather than the CKD EPI formula, which was utilized by the CAREHES and SardiNIA studies. After all, the impact of the CKD-EPI equation on CKD diagnosis was highlighted by the INCIPE study authors, who subsequently commented that its use modifies the results, especially in the early stages of the disease [60]. As also suggested by a 2018 meta-analysis, which found no significant differences between the two formulas in patients with a eGFR of less than 60 mL/min/1.73 m^2^ [61]. Thus, the estimation of CKD G3-5 should not be gravitated by significant interference related to the use of the MDRD formula. On the contrary, the number of CKD G1-2 patients should be underestimated using the MDRD formula, which partially explains the discrepancy in CKD G1-2 estimation between INCIPE and CAREHES or SardiNIA studies.

Considering the issues with the formula used in estimating CKD, we would like to highlight a problem in the classification of CKD according to the CKD-EPI formula in patients over 80 years old, as this equation has not been validated in this age group [62,63]. Consequently, the CKD-EPI formula may underestimate the true prevalence of CKD in an increasing number of patients. As nephrologists are aware, biomarkers of kidney disease can have varying meanings depending on the clinical context. Therefore, the reliability of creatinine, NGAL, Cystatin C, and copeptin in estimating kidney function depends on the validation context. In other words, an increase in creatinine in a young bodybuilder does not always indicate kidney failure [64,65], a rise in NGAL is not always related to acute kidney failure [66,67], interpreting Cystatin C requires understanding of pharmacological treatments [68,69], and copeptin appears to be a reliable marker of kidney function in autosomal dominant polycystic kidney disease but has not been tested in other disorders [70,71,72,73]. Indeed, old patients present a high risk of misclassification of CKD due to sarcopenia and its impact on the creatinine levels [74,75]. As the example of Brain Natriuretic Peptide demonstrates, age can significantly influence biomarker reference values [76,77,78], transforming an abnormal value in younger age classes into a normal value in older individuals.

Despite the difference, the projection of the number of CKD patients remains impressive, also in the CAREHES and SardiNIA studies [28,29], which concur in testifying to an increase in the number of older adults with CKD, particularly in the most severe stage of CKD. The management of CKD in the general population cannot overlook this evidence to define the priority care necessary to guarantee the best possible treatment for every patient. In the near future, nephrologists will face a high number of patients with limited personnel resources, as the number of nephrologists and nurses appears inadequate to support the increasing number of CKD patients. From an ethical perspective, defining the priority of nephrology care is essential to avoid discrimination between patients and to limit the opportunity for undertreating patients with more severe conditions, thereby favoring those with less severe conditions. Although ideally, all CKD patients merit nephrological care to limit the progression of the chronic disease, the limitation of available resources raises a question of care priority. Our district could serve as an example. The number of nephrologists is insufficient to manage all CKD patients. Therefore, it is reasonable to prioritize the management of high- and very high-risk patients over those with low- and moderate-risk CKD, while also ensuring that patients who require screening or annual control receive the necessary attention. In this scenario, the collaboration with a general practitioner (GP) could be a good compromise. In Italy, the number of patients for each GP is a maximum of 1800, so each GP should have around 160 CKD patients with low and moderate risk of progression, according to the INCIPE study reports. If the GP can easily follow the screening phase and first treatment advice in G1A1 and G2A1, active surveillance and treatment of G1A2, G2A2, and G3aA1 could be more difficult. We must remember that moderate-risk CKD patients usually do not experience CKD complications such as anemia, metabolic acidosis, impairment of calcium and phosphate balance, or electrolyte abnormalities, which typically appear in the later stages of kidney disease and require a nephrologist’s expertise. Obviously, the involvement of GPs in the management of CKD should be supported by several strategies, such as active training in a nephrology department during the education period and the implementation of remote consultation. This collaboration not only aids in chronic conditions but also in acute settings, where the expertise of the nephrologist can significantly impact diagnostic and therapeutic approaches, particularly in the context of rare diseases in the nephrology area [79,80,81,82,83]. Establishing a direct communication channel between the GP and the nephrologist has beneficial effects for all parties involved in the process. The GP would reduce the time dedicated to CKD, limiting its activity for patients starting surveillance and basal treatment, leaving the care of high- and very-high-risk patients to the nephrologist. The nephrologist would limit the number of consultations, allowing for a more effective allocation of time and resources to severe CKD patients, who specifically require nephrology expertise. The patient would have an adequate answer in all possible phases of his disease.

Furthermore, the use of telemedicine for nephrology evaluation, supported by nurses, appears to be another way to limit the need for hospital visits in both elderly and younger individuals, thereby improving quality of life [84,85,86,87,88] and reducing management costs [89]. Finally, considering the possible underestimation of the number of CKD G1-2 and the limited resources of the GP, an informative campaign about kidney disease could help raise awareness among the general population, allowing them to understand its risk factors, symptoms, and the adequate behavior to limit its impact. National and international events, such as World Kidney Day, as well as local events related to nephrology issues, are effective in sensitizing the general population. However, a targeted campaign in high schools could be more helpful in preventing CKD.

A specific remark merits the patients who reach CKD G5. As reported in the CKDG5 Vicenza study [52] and confirmed by the CAREHES and SardiNIA studies [28,29], the number of these patients increases with the aging of the population. According to our district’s projections, the number of patients likely to have CKD G5 exceeded twice the available dialysis capacity. Indeed, renal replacement therapy is not always the best option for all patients in terms of survival and quality of life [90]. In older patients with higher comorbidity scores and in very old patients with a valid urine output, comprehensive conservative management can be the best option, as it limits the impact on quality of life without a proven detrimental effect on survival.

Moreover, the introduction of home dialysis could present a promising solution for managing CKD G5 patients with dialysis needs, potentially reducing costs and the demand for personnel resources. Notably, peritoneal dialysis has shown a comparable survival curve to that of hemodialysis patients, with a likely enhancement in quality of life. Despite persistent advocacy in recent years, peritoneal dialysis remains underutilized [91,92,93]. In light of the challenges ahead, nephrologists should consider a dual approach, incorporating both comprehensive conservative management and home dialysis, to improve the outlook for CKD G5 patients.

Our report has several limitations. Firstly, the estimation of patient numbers and outpatient clinic controls is closely tied to the chosen epidemiology data. This limitation could not be overcome unless a new epidemiology study is conducted in the same area of the projection. Secondly, the INCIPE study is somewhat dated. It may underestimate the actual impact of CKD in our district, particularly when considering the changes in the prevalence of the main comorbidities (diabetes, hypertension, and obesity) between 2006 and the present. According to the Istituto Superiore di Sanità (ISS) report, in Veneto, a reduction in hypertension prevalence was accompanied by an increase in the prevalence of diabetes and obesity [94,95,96]. However, no other Italian study on CKD prevalence has been conducted in Veneto, and the other studies, CAREHES and SardiNIA, were performed during a comparable period. Despite this limitation, we hope the critical evaluation of the results and comparison with other projections have minimized this issue. Thirdly, the MDRD formula underestimates the CKD G1-2 patients, who can be significantly more than our projection. Finally, speculation about the Padua Healthcare district may seem only of local relevance and not valuable for other districts or areas worldwide. This statement is valid only partially. If the application of epidemiological data to other realities can yield different numbers regarding CKD patients and raise additional considerations about resources and care priorities, evaluating the impact of the changed prevalence of CKD in a specific reality serves as an incentive to examine the matter and find reliable solutions in other realities as well.

## 5. Conclusions

Previous epidemiology reports on CKD have shown varying prevalence numbers across different stages, but collectively indicate an increasing number of older patients and insufficient personnel resources. The projection of CKD numbers in the Padua Healthcare district, according to the INCIPE study, reveals a significant gap between the number of potential patients and the current number of nephrologists, indicating a need for nephrologists in outpatient clinics that is over four to six times the current number. Addressing this disparity requires immediate action to ensure adequate care for all CKD patients. Given the priority care, medical evidence, and the potential personnel resources to allocate in the outpatient clinic, a stronger collaboration between the GP and the nephrologist is highly recommended. Establishing a dynamic structure to facilitate this collaboration would enable patients to receive timely care at all stages of kidney disease. Furthermore, in the management of CKD G5, nephrologists should conduct a comprehensive evaluation of each patient to ensure the best possible treatment, tailored to their comorbidities, age, and preferences.

## Figures and Tables

**Table 1:** CKD G1-4 prevalence according to INCIPE study results.

	CKD 1	CKD2	CKD 3a	CKD3b	CKD 4	CKD 1 to 4 Stages
Total	1.7%	4.3%	5.1%	1.3%	0.3%	12.7%
Men	1.7%	5.0%	4.8%	1.4%	0.3%	13.2%
Female	1.6%	3.7%	5.4%	1.3%	0.2%	12.2%
40–49 years	1.8%	0.7%	0.3%	0.1%	0.0%	2.9%
50–59 years	2.0%	1.7%	1.5%	0.1%	0.0%	5.3%
60–69 years	2.0%	5.7%	3.8%	0.5%	0.3%	12.3%
70–89 years	0.7%	9.2%	13.5%	4.2%	0.7%	28.2%
>80 years	0.0%	11.8%	29.2%	9.3%	1.9%	52.2%

**Table 2:** Estimated prevalence of CKD stages according to eGFR and albuminuria in classes of age.

	G1 *			G2 *			G3 *			G4 *			G5 **		
%	1.7			4.3			5.1			0.3			0.2		
	A1	A2	A3	A1	A2	A3	A1	A2	A3	A1	A2	A3	A1	A2	A3
%	0.088	0.790	0.122	0.063	0.799	0.138	0.794	0.149	0.057	0.517	0.172	0.431	0.2		

Footnotes: G1: eGFR > 90 mL/min/1.73m^2^, G2: 89 > eGFR > 60 mL/min/1.73m^2^, G3 59 > eGFR > 30 mL/min/1.73m^2^, G4: 29 > eGFR > 15 mL/min/1.73m^2^, G5: eGFR < 15 mL/min/1.73m^2^, A1 < 30 mg/g Creatinine, 30 < A2 < 299 mg/g Creatinine, A3 > 300 mg/g Creatinine. Green represents the low-risk class, yellow moderate risk, orange high risk, red and dark red very high risk with 3 or 4 controls, respectively. * The prevalence estimated by the INCIPE study, ** the prevalence estimated by the CKD G5 Vicenza study.

**Table 3:** Class of age prevalence in the CKD G5 population according to the Vicenza study.

Age	Prevalence
40–49 years	4.2%
50–59 years	8.7%
60–69 years	13.9%
70–79 years	26.4%
80–89 years	34.9%
>90 years	9.4%

**Table 4:** Number of suggested nephrologist consultations by KDIGO guidelines in one year.

	G1			G2			G3a			G3b			G4			G5		
	A1	A2	A3	A1	A2	A3	A1	A2	A3	A1	A2	A3	A1	A2	A3	A1	A2	A3
Number of controls	1	1	3	1	1	3	1	2	3	2	3	3	3	3	+4	+4		

Footnotes. G1: eGFR > 90 mL/min/1.73m^2^, G2: 89 > eGFR > 60 mL/min/1.73m^2^, G3a: 59 > eGFR > 45 mL/min/1.73m^2^, G3b: 44 > eGFR3 > 30 mL/min/1.73m^2^, G4: 29 > eGFR > 15 mL/min/1.73m^2^, G5: eGFR < 15 mL/min/1.73m^2^, A1 < 30 mg/g Creatinine, 30 < A2 < 299 mg/g Creatinine, A3 > 300 mg/g Creatinine. Green represents the low-risk class, yellow moderate risk, orange high risk, and red very high risk.

**Table 5:** Number of patients and controls in Padua Healthcare District.

	A1	A2	A3	Cumulative
G1	359 **(359)**	3228 **(3228)**	497 **(993)**	4084 **(4581)**
G2	646 **(646)**	8257 **(8257)**	1428 **(2856)**	10,331 **(11,759)**
G3a	9730 **(9730)**	1830 **(3661)**	693 **(407)**	12,253 **(15,469)**
G3b	2480 **(4960)**	467 **(1400)**	177 **(530)**	3123 **(6890)**
G4	372 **(1117)**	124 **(1242)**	1310 **(4138)**	807 **(2132)**
G5 no RRT				340 **(1360)**
G1-5 no RRT				30,939 **(42,790)**

Footnotes: Number and **(Number)** refer to the number of patients and number of controls, respectively. G1: eGFR > 90 mL/min/1.73m^2^, G2: 89 > eGFR > 60 mL/min/1.73m^2^, G3a: 59 > eGFR > 45 mL/min/1.73m^2^, G3b: 44 > eGFR3 > 300 mL/min/1.73m^2^, G4: 29 > eGFR > 15 mL/min/1.73m^2^, G5: eGFR < 15 mL/min/1.73m^2^, A1 < 30 mg/g Creatinine, 30 < A2 < 299 mg/g Creatinine, A3 > 300 mg/g Creatinine, RRT renal replacement therapy.

## Data Availability

These data were derived from the following resources available in the public domain: https://www.tuttitalia.it/veneto/provincia-di-padova/statistiche/popolazione-eta-sesso-stato-civile-2024/, accessed on 15 April 2025.

## Abbreviations

The following abbreviations are used in this manuscript:

CKD	Chronic Kidney Disease
eGFR	estimated Glomerular Filtration Rate
INCIPE	Initiative on Nephropathy, of relevance to public health, which is Chronic, possibly in its Initial stages, and carries a Potential risk of major clinical Endpoints
CAREHES	Cardiovascular risk in Renal Patients of the Italian Health Examination Survey
SGLT2	Sodium-Glucose Cotransporter-2
MRA	Mineralocorticoid Receptor Antagonist
GLP-1 RA	Glucagon-like peptide-1 Receptor Agonists
ISTAT	Italian Statistics Institute
RRT	Renal Replacement Therapy
GP	General Practitioner

## References

[1] Vanholder R., Annemans L., Brown E., Gansevoort R., Gout-Zwart J.J., Lameire N., Morton R.L., Oberbauer R., Postma M.J., Tonelli M. (2017). Reducing the costs of chronic kidney disease while delivering quality health care: A call to action. Nat. Rev. Nephrol..

[2] Janse R.J., Milders J., Rotmans J.I., Caskey F.J., Evans M., Torino C., Szymczak M., Drechsler C., Wanner C., Pippias M. (2025). Predicting Hospitalization and Related Outcomes in Advanced Chronic Kidney Disease: A Systematic Review, External Validation, and Development Study. Kidney Med..

[3] GBD 2021 Causes of Death Collaborators (2024). Global burden of 288 causes of death and life expectancy decomposition in 204 countries and territories and 811 subnational locations, 1990–2021: A systematic analysis for the Global Burden of Disease Study 2021. Lancet.

[4] GBD 2021 Risk Factors Collaborators (2024). Global burden and strength of evidence for 88 risk factors in 204 countries and 811 subnational locations, 1990–2021: A systematic analysis for the Global Burden of Disease Study 2021. Lancet.

[5] Go A.S., Chertow G.M., Fan D., McCulloch C.E., Hsu C.Y. (2004). Chronic kidney disease and the risks of death, cardiovascular events, and hospitalization. N. Engl. J. Med..

[6] Van der Velde M., Matsushita K., Coresh J., Astor B.C., Woodward M., Levey A., de Jong P., Gansevoort R.T., Chronic Kidney Disease Prognosis Consortium (2011). Lower estimated glomerular filtration rate and higher albuminuria are associated with all-cause and cardiovascular mortality: A collaborative meta-analysis of high-risk population cohorts. Kidney Int..

[7] GBD Chronic Kidney Disease Collaboration (2020). Global, regional, and national burden of chronic kidney disease, 1990–2017: A systematic analysis for the Global Burden of Disease Study 2017. Lancet.

[8] Tungsanga S., Fung W., Okpechi I.G., Ye F., Ghimire A., Li P.K.-T., Shlipak M.G., Tummalapalli S.L., Arruebo S., Caskey F.J. (2024). Organization and Structures for Detection and Monitoring of CKD Across World Countries and Regions: Observational Data From a Global Survey. Am. J. Kidney Dis..

[9] Kalyesubula R., Conroy A.L., Calice-Silva V., Kumar V., Onu U., Batte A., Kaze F.F., Fabian J., Ulasi I. (2022). Screening for Kidney Disease in Low- and Middle-Income Countries. Semin. Nephrol..

[10] https://www.cdc.gov/kidney-disease/php/data-research/index.html.

[11] https://kidney.ca/KFOC/media/images/PDFs/Facing-the-Facts-2023-HIghlights-from-the-Annual-Statistics-on-Organ-Donation.pdf.

[12] Nagai K., Asahi K., Iseki K., Yamagata K. (2021). Estimating the prevalence of definitive chronic kidney disease in the Japanese general population. Clin. Exp. Nephrol..

[13] Mehta K., Randall S., Lee C.M.Y., Thomas E., Chakera A., Chai K., Estai M., Frith M., Hendrie D., Boyd J. (2025). Prevalence of chronic kidney disease in Western Australia, 2010–2020. BMJ Open.

[14] Brück K., Stel V.S., Gambaro G., Hallan S., Völzke H., Ärnlöv J., Kastarinen M., Guessous I., Vinhas J., Stengel B. (2016). CKD Prevalence Varies across the European General Population. J. Am. Soc. Nephrol..

[15] Jenssen T.G., Bodegård J., Sveen K.A., Thuresson M., Birkeland K.I. (2025). Prevalence, outcomes, costs, and treatments of a contemporary population with chronic kidney disease in Norway: A nationwide observational study. BMC Nephrol..

[16] Escobar C., Palacios B., Aranda U., Capel M., Sicras A., Sicras A., Hormigo A., Alcázar R., Manito N., Botana M. (2021). Costs and healthcare utilisation of patients with chronic kidney disease in Spain. BMC Health Serv. Res..

[17] Escobar C., Aranda U., Palacios B., Capel M., Sicras A., Sicras A., Hormigo A., Alcázar R., Manito N., Botana M. (2021). Epidemiology, clinical profile, management, and two-year risk complications among patients with chronic kidney disease in Spain. Nefrol. Engl. Ed..

[18] Stack A.G., Casserly L.F., Cronin C.J., Chernenko T., Cullen W., Hannigan A., Saran R., Johnson H., Browne G., Ferguson J.P. (2014). Prevalence and variation of Chronic Kidney Disease in the Irish health system: Initial findings from the National Kidney Disease Surveillance Programme. BMC Nephrol..

[19] Gifford F.J., Methven S., Boag D.E., Spalding E.M., Macgregor M.S. (2011). Chronic kidney disease prevalence and secular trends in a UK population: The impact of MDRD and CKD-EPI formulae. QJM Int. J. Med..

[20] Aumann N., Baumeister S.E., Rettig R., Lieb W., Werner A., Döring A., Peters A., Koenig W., Hannemann A., Wallaschofski H. (2015). Regional variation of chronic kidney disease in Germany: Results from two population-based surveys. Kidney Blood Press. Res..

[21] Gambaro G., Yabarek T., Graziani M.S., Gemelli A., Abaterusso C., Frigo A.C., Marchionna N., Citron L., Bonfante L., Grigoletto F. (2010). Prevalence of CKD in northeastern Italy: Results of the INCIPE study and comparison with NHANES. Clin. J. Am. Soc. Nephrol..

[22] Guarda L., Bozzeda A.L., Ricci P. (2010). Epidemiologia della insufficienza renale cronica in una popolazione: I residenti nella provincia di Mantova [Epidemiology of chronic kidney disease in the population of the Mantua district]. G. Ital. Nefrol..

[23] Gorini A., Costanzo A.M., Egan C.G., di Luzio Paparatti U. (2012). Renal status in adult volunteers in central Italy: Results from Family Abbott Renal Disease Monitoring Project (FARM) study. J. Nephrol..

[24] Donfrancesco C., Palleschi S., Palmieri L., Rossi B., Noce C.L., Pannozzo F., Spoto B., Tripepi G., Zoccali C., Giampaoli S. (2013). Estimated glomerular filtration rate, all-cause mortality and cardiovascular diseases incidence in a low risk population: The MATISS study. PLoS ONE.

[25] Ciao G., Berardi P., Vecchi L., Buoncristiani E., Eroli M., Levêque A. (2014). Epidemiologia della malattia renale cronica in una popolazione: I residenti nel territorio umbro “eugubino-gualdese” [Epidemiology of chronic kidney disease in a population: Residents in the territory of Umbria “Gubbio—Gualdese”]. G. Ital. Nefrol..

[26] Marino C., Ferraro P.M., Bargagli M., Cascini S., Agabiti N., Gambaro G., Davoli M. (2020). Prevalence of chronic kidney disease in the Lazio region, Italy: A classification algorithm based on health information systems. BMC Nephrol..

[27] Capuano V., Lamaida N., Borrelli M.I., Capuano E., Fasolino A., Capuano E., Sonderegger M., Capuano R., Citro V., Franculli F. (2012). Prevalenza e trend (1998–2008) dell’insufficienza renale cronica in un’area dell’Italia meridionale: I dati del progetto VIP [Chronic kidney disease prevalence and trends (1998–2008) in an area of southern Italy. The data of the VIP project]. G. Ital. Nefrol..

[28] Pani A., Bragg-Gresham J., Masala M., Piras D., Atzeni A., Pilia M.G., Ferreli L., Balaci L., Curreli N., Delitala A. (2014). Prevalence of CKD and its relationship to eGFR-related genetic loci and clinical risk factors in the SardiNIA study cohort. J. Am. Soc. Nephrol..

[29] De Nicola L., Donfrancesco C., Minutolo R., Lo Noce C., Palmieri L., De Curtis A., Iacoviello L., Zoccali C., Gesualdo L., Conte G. (2015). Prevalence and cardiovascular risk profile of chronic kidney disease in Italy: Results of the 2008–12 National Health Examination Survey. Nephrol Dial Transplant..

[30] Airoldi C., Pagnoni F., Cena T., Ceriotti D., De Ambrosi D., De Vito M., Faggiano F. (2023). Estimate of the prevalence of subjects with chronic diseases in a province of Northern Italy: A retrospective study based on administrative databases. BMJ Open..

[31] Brück K., Jager K.J., Dounousi E., Kainz A., Nitsch D., Ärnlöv J., Rothenbacher D., Browne G., Capuano V., Ferraro P.M. (2015). Methodology used in studies reporting chronic kidney disease prevalence: A systematic literature review. Nephrol. Dial. Transplant..

[32] Stevens P.E., Ahmed S.B., Carrero J.J., Foster B., Francis A., Hall R.K., Herrington W.G., Hill G., Inker L.A., Kazancıoğlu R. (2024). Kidney Disease: Improving Global Outcomes (KDIGO) CKD Work Group. KDIGO 2024 Clinical Practice Guideline for the Evaluation and Management of Chronic Kidney Disease. Kidney Int..

[33] Iatridi F., Carrero J.J., Cornec-Le Gall E., Kanbay M., Luyckx V., Shroff R., Ferro C.J. (2025). KDIGO 2024 Clinical Practice Guideline for the Evaluation and Management of Chronic Kidney Disease in Children and Adults: A commentary from the European Renal Best Practice (ERBP). Nephrol Dial Transplant..

[34] Zhang N., Shi J. (2025). Domain-specific physical activity and sedentary behavior in relation to chronic kidney disease: A cross-sectional analysis of 24,950 U.S. adults in NHANES 1999–2012. Ren Fail..

[35] Zhang L., Liu F., Li M., Fan Y. (2025). Sedentary behavior and risk of chronic kidney disease: A systematic review and meta-analysis. Int. Urol. Nephrol..

[36] Battaglia Y., Baciga F., Bulighin F., Amicone M., Mosconi G., Storari A., Brugnano R., Pozzato M., Motta D., D’alessandro C. (2024). Physical activity and exercise in chronic kidney disease: Consensus statements from the Physical Exercise Working Group of the Italian Society of Nephrology. J. Nephrol..

[37] Kaimori J.Y., Sakaguchi Y., Oka T., Isaka Y. (2025). Plant-Dominant Low-Protein Diets: A Promising Dietary Strategy for Mitigating Disease Progression in People with Chronic Kidney Disease-A Comprehensive Review. Nutrients.

[38] Martino F.K., Zattarin A., Cinquini C., Toniazzo S., Francini Pesenti F., Stefanelli L.F., Cacciapuoti M., Bettin E., Calò L.A., Spinella P. (2024). Low-Protein Diet in Elderly Patients with Chronic Kidney Disease Stage 4 and 5 in Conservative Management: Focus on Sarcopenia Development. Nutrients.

[39] Amiri Khosroshahi R., Zare M., Zeraattalab-Motlagh S., Kiany F., Talebi S., Mohammadi H. (2025). Effects of a Low-Protein Diet on Kidney Function in Patients with Chronic Kidney Disease: An Umbrella Review of Systematic Reviews and Meta-analyses of Randomized Controlled Trials. Nutr. Rev..

[40] Wakino S., Hasegawa K., Tamaki M., Minato M., Inagaki T. (2025). Kidney-Gut Axis in Chronic Kidney Disease: Therapeutic Perspectives from Microbiota Modulation and Nutrition. Nutrients.

[41] Martino F.K., Stefanelli L.F., Zattarin A., Lovato Correa Dias L., Redi G., Khalf R., Del Prete D., Nalesso F. (2025). Protein Counting as an Educational Strategy to Optimize Low-Protein-Diet Adherence and Satisfaction in Stage 4 and 5 Chronic Kidney Disease Patients: A Pilot Study. Nutrients.

[42] Marrone G., Di Lauro M., Cornali K., Masci C., Vanni G., Vita C., Noce A. (2025). Sustainability and role of plant-based diets in chronic kidney disease prevention and treatment. Front. Pharmacol..

[43] Wu L., Ma W., Zhang H., Yang T., Sun M., Yang Z., Guo X. (2025). Effect of intensive water-salt diet nursing intervention on blood pressure and volume load in patients with chronic renal failure. Ren. Fail..

[44] He Y., Li J., Rao J., Lai W., Wei Q., Li H., Li Y., Peng H., Zhang J. (2025). Joint effects of sodium intake and circadian rhythm of urinary sodium excretion on prognosis of chronic kidney disease: A prospective study. Ther. Adv. Chronic Dis..

[45] Herrington W.G., Staplin N., Wanner C., Green J.B., Hauske S.J., Emberson J.R., Preiss D., Judge P., Mayne K.J., The EMPA-KIDNEY Collaborative Group (2023). Empagliflozin in patients with chronic kidney disease. N. Engl. J. Med..

[46] Nuffield Department of Population Health Renal Studies Group (2022). SGLT Inhibitor Meta-Analysis Cardio-Renal Trialists’ Consortium. Impact of diabetes on the effects of sodium glucose co-transporter-2 inhibitors on kidney outcomes: Collaborative meta-analysis of large placebo controlled trials. Lancet.

[47] Staplin N., Roddick A.J., Emberson J., Reith C., Riding A., Wonnacott A., Kuverji A., Bhandari S., Baigent C., Haynes R. (2021). Net effects of sodium-glucose co-transporter-2 inhibition in different patient groups: A meta-analysis of large placebo-controlled randomized trials. eClinicalMedicine.

[48] Heerspink H.J.L., Stefánsson B.V., Correa-Rotter R., Chertow G.M., Greene T., Hou F.F., Mann J.F.E., McMurray J.J.V., Lindberg M., Rossing P. (2020). Dapagliflozin in Patients with Chronic Kidney Disease. N. Engl. J. Med..

[49] Perkovic V., Jardine M.J., Neal B., Bompoint S., Heerspink H.J.L., Charytan D.M., Edwards R., Agarwal R., Bakris G., Bull S. (2019). Canagliflozin and Renal Outcomes in Type 2 Diabetes and Nephropathy. N. Engl. J. Med..

[50] Cherney D.Z.I., Dekkers C.C.J., Barbour S.J., Cattran D., Abdul Gafor A.H., Greasley P.J., Laverman G.D., Lim S.K., Di Tanna G.L., Reich H.N. (2020). Effects of the SGLT2 inhibitor dapagliflozin on proteinuria in non-diabetic patients with chronic kidney disease (DIAMOND): A randomised, double-blind, crossover trial. Lancet Diabetes Endocrinol..

[51] Currie G., Taylor A.H., Fujita T., Ohtsu H., Lindhardt M., Rossing P., Boesby L., Edwards N.C., Ferro C.J., Townend J.N. (2016). Effect of mineralocorticoid receptor antagonists on proteinuria and progression of chronic kidney disease: A systematic review and meta-analysis. BMC Nephrol..

[52] Bakris G.L., Agarwal R., Anker S.D., Pitt B., Ruilope L.M., Rossing P., Kolkhof P., Nowack C., Schloemer P., Joseph A. (2020). Effect of Finerenone on Chronic Kidney Disease Outcomes in Type 2 Diabetes. N. Engl. J. Med..

[53] Rossing P., Baeres F.M.M., Bakris G., Bosch-Traberg H., Gislum M., Gough S.C.L., Idorn T., Lawson J., Mahaffey K.W., Mann J.F.E. (2023). The rationale, design and baseline data of FLOW, a kidney outcomes trial with once-weekly semaglutide in people with type 2 diabetes and chronic kidney disease. Nephrol. Dial. Transplant..

[54] Martino F.K., Fanton G., Zanetti F., Carta M., Nalesso F., Novara G. (2024). Stage 5 Chronic Kidney Disease: Epidemiological Analysis in a NorthEastern District of Italy Focusing on Access to Nephrological Care. J. Clin. Med..

[55] Oliveira Coelho F., Andrade L. (2021). Smoking and Kidney Disease: Risk Factors, Challenges, and Preventive Strategies. Contrib. Nephrol..

[56] Lee S., Kang S., Joo Y.S., Lee C., Nam K.H., Yun H.R., Park J.T., Chang T.I., Yoo T.H., Kim S.W. (2021). Smoking, Smoking Cessation, and Progression of Chronic Kidney Disease: Results from KNOW-CKD Study. Nicotine Tob. Res..

[57] Jo W., Lee S., Joo Y.S., Nam K.H., Yun H.R., Chang T.I., Kang E.W., Yoo T.H., Han S.H., Kang S.W. (2020). Association of smoking with incident CKD risk in the general population: A community-based cohort study. PLoS ONE.

[58] Moeinzadeh F., Shahidi S., Seirafian S., Rouhani M.H., Mortazavi M., Maghami-Mehr A., Vahdat S. (2023). Association of alcohol consumption with the prevalence and various stages of chronic kidney disease. J. Res. Med. Sci..

[59] Joo Y.S., Koh H., Nam K.H., Lee S., Kim J., Lee C., Yun H.R., Park J.T., Kang E.W., Chang T.I. (2020). Alcohol Consumption and Progression of Chronic Kidney Disease: Results from the Korean Cohort Study for Outcome in Patients with Chronic Kidney Disease. Mayo Clin. Proc..

[60] Li Y., Zhu B., Song N., Shi Y., Fang Y., Ding X. (2022). Alcohol consumption and its association with chronic kidney disease: Evidence from a 12-year China health and Nutrition Survey. Nutr. Metab. Cardiovasc. Dis..

[61] McFadden E.C., Hirst J.A., Verbakel J.Y., McLellan J.H., Hobbs F.D.R., Stevens R.J., O’Callaghan C.A., Lasserson D.S. (2018). Systematic Review and Metaanalysis Comparing the Bias and Accuracy of the Modification of Diet in Renal Disease and Chronic Kidney Disease Epidemiology Collaboration Equations in Community-Based Populations. Clin. Chem..

[62] Ferraro P.M., Lombardi G., Gambaro G. (2022). Impact of the new, race-free CKD-EPI equation on prevalence and clinical outcomes of CKD in northeastern Italy: The INCIPE study. J. Nephrol..

[63] Levey A.S., Stevens L.A., Schmid C.H., Zhang Y.L., Castro A.F., Kusek J.W., Eggers P., Van Lente F., Greene T. (2009). A new equation to estimate glomerular filtration rate. Ann. Intern. Med..

[64] Levey A.S., Inker L.A., Coresh J. (2014). GFR estimation: From physiology to public health. Am. J. Kidney Dis..

[65] Velema M.S., de Ronde W. (2011). Elevated plasma creatinine due to creatine ethyl ester use. Neth. J. Med..

[66] Williamson L., New D. (2014). How the use of creatine supplements can elevate serum creatinine in the absence of underlying kidney pathology. BMJ Case Rep..

[67] Bolignano D., Coppolino G., Lacquaniti A., Buemi M. (2010). From kidney to cardiovascular diseases: NGAL as a biomarker beyond the confines of nephrology. Eur. J. Clin. Investig..

[68] Martino F.K., Filippi I., Giavarina D., Kaushik M., Rodighiero M.P., Crepaldi C., Teixeira C., Nadal A.F., Rosner M.H., Ronco C. (2012). Neutrophil gelatinase-associated lipocalin in the early diagnosis of peritonitis: The case of neutrophil gelatinase-associated lipocalin. Contrib. Nephrol..

[69] Onopiuk A., Tokarzewicz A., Gorodkiewicz E. (2015). Cystatin C: A kidney function biomarker. Adv. Clin. Chem..

[70] van Eeghen S.A., Wiepjes C.M., T’Sjoen G., Nokoff N.J., den Heijer M., Bjornstad P., van Raalte D.H. (2023). Cystatin C-Based eGFR Changes during Gender-Affirming Hormone Therapy in Transgender Individuals. Clin. J. Am. Soc. Nephrol..

[71] Westhuyzen J. (2006). Cystatin C: A promising marker and predictor of impaired renal function. Ann. Clin. Lab. Sci..

[72] Corradi V., Martino F., Gastaldon F., Scalzotto E., Caprara C., Fortunato A., Pinaffo G., Marchetti C., Fabbi F., Giavarina D. (2016). Copeptin levels and kidney function in ADPKD: Case-control study. Clin. Nephrol..

[73] Corradi V., Gastaldon F., Caprara C., Giuliani A., Martino F., Ferrari F., Ronco C. (2017). Predictors of rapid disease progression in autosomal dominant polycystic kidney disease. Minerva Med..

[74] Arjune S., Oehm S., Todorova P., Gansevoort R.T., Bakker S.J.L., Erger F., Benzing T., Burst V., Grundmann F., Antczak P. (2023). Copeptin in autosomal dominant polycystic kidney disease: Real-world experiences from a large prospective cohort study. Clin. Kidney J..

[75] Gazi G., Cruciat R.C., Leucuta D.C., Al Srouji N., Popa S.L., Ismaiel M., Dumitrascu D.I., Ismaiel A. (2025). Copeptin as a Biomarker in Chronic Kidney Disease-A Systematic Review and Meta-Analysis. Biomolecules.

[76] da Silva Selistre L., Rech D.L., de Souza V., Iwaz J., Lemoine S., Dubourg L. (2019). Diagnostic Performance of Creatinine-Based Equations for Estimating Glomerular Filtration Rate in Adults 65 Years and Older. JAMA Intern. Med..

[77] Castel-Branco M.M., Lavrador M., Cabral A.C., Pinheiro A., Fernandes J., Figueiredo I.V., Fernandez-Llimos F. (2024). Discrepancies among equations to estimate the glomerular filtration rate for drug dosing decision making in aged patients: A cross sectional study. Int. J. Clin. Pharm..

[78] Keyzer J.M., Hoffmann J.J., Ringoir L., Nabbe K.C., Widdershoven J.W., Pop V.J. (2014). Age- and gender-specific brain natriuretic peptide (BNP) reference ranges in primary care. Clin. Chem. Lab. Med..

[79] Crepaldi C., Rosner M., Teixeira C., Martos L.B., Martino F.K., Rodighiero M.P., Ronco C. (2014). Is brain natriuretic peptide a reliable biomarker of hydration status in all peritoneal dialysis patients?. Blood Purif..

[80] Alehagen U., Goetze J.P., Dahlström U. (2007). Reference intervals and decision limits for B-type natriuretic peptide (BNP) and its precursor (Nt-proBNP) in the elderly. Clin. Chim. Acta..

[81] Milan Manani S., Virzì G.M., Giuliani A., Clementi A., Brocca A., Dissegna D., Martino F., d’Amore E.S.G., Ronco C. (2017). Hemolytic Uremic Syndrome and Kidney Transplantation: A Case Series and Review of the Literature. Nephron.

[82] Linares D., Luna B., Loayza E., Taboada G., Ramaswami U. (2023). Prevalence of Fabry disease in patients with chronic kidney disease: A systematic review and meta-analysis. Mol. Genet. Metab..

[83] Heiderscheit A.K., Hauer J.J., Smith R.J.H. (2022). C3 glomerulopathy: Understanding an ultra-rare complement-mediated renal disease. Am. J. Med. Genet. C Semin. Med. Genet..

[84] Sahay M., Gowrishankar S. (2010). Glomerulocystic disease. NDT Plus..

[85] Caprara C., Kinsey G.R., Corradi V., Xin W., Ma J.Z., Scalzotto E., Martino F.K., Okusa M.D., Nalesso F., Ferrari F. (2016). The Influence of Hemodialysis on T Regulatory Cells: A Meta-Analysis and Systematic Review. Blood Purif..

[86] Jung H.Y., Jeon Y., Kim Y.S., Kim D.K., Lee J.P., Yang C.W., Ko E.J., Ryu D.-R., Kang S.-W., Park J.T. (2021). Outcomes of Remote Patient Monitoring for Automated Peritoneal Dialysis: A Randomized Controlled Trial. Nephron.

[87] Ghozali M.T., Satibi S., Forthwengel G. (2023). The impact of mobile health applications on the outcomes of patients with chronic kidney disease: A systematic review and meta-analysis. J. Med. Life.

[88] Zhu J., Xie J., Luo Y., Dong X., Lu Z., Zhang H., Wang J., Liu M., Cheng A.S. (2025). The Impact of Digital Health Interventions on Psychological Health, Self-Efficacy, and Quality of Life in Patients With End-Stage Kidney Disease: Systematic Review and Meta-Analysis. J. Med. Internet Res..

[89] Amici G., D’Angela D., Lo Cicero A., Romanini D., Martino F.K., Spandonaro F. (2021). Pilot health technology assessment study: Organizational and economic impact of remote monitoring system for home automated peritoneal dialysis. Int. Urol. Nephrol..

[90] Martino F.K., Novara G., Nalesso F., Calò L.A. (2023). Conservative Management in End-Stage Kidney Disease between the Dialysis Myth and Neglected Evidence-Based Medicine. J. Clin. Med..

[91] Li P.K., Chow K.M., Van de Luijtgaarden M.W., Johnson D.W., Jager K.J., Mehrotra R., Naicker S., Pecoits-Filho R., Yu X.Q., Lameire N. (2017). Changes in the worldwide epidemiology of peritoneal dialysis. Nat. Rev. Nephrol..

[92] Jain A.K., Blake P., Cordy P., Garg A.X. (2012). Global trends in rates of peritoneal dialysis. J. Am. Soc. Nephrol..

[93] Liu F.X., Gao X., Inglese G., Chuengsaman P., Pecoits-Filho R., Yu A. (2015). A Global Overview of the Impact of Peritoneal Dialysis First or Favored Policies: An Opinion. Perit. Dial. Int..

[94] https://www.cuore.iss.it/indagini/CuoreData.

[95] https://www.epicentro.iss.it/diabete/epidemiologia-italia.

[96] https://www.epicentro.iss.it/obesita/epidemiologia-italia.

